# Satellite cell self‐renewal in endurance exercise is mediated by inhibition of mitochondrial oxygen consumption

**DOI:** 10.1002/jcsm.12601

**Published:** 2020-08-03

**Authors:** Phablo Abreu, Alicia J. Kowaltowski

**Affiliations:** ^1^ Departamento de Bioquímica, Instituto de Química Universidade de São Paulo São Paulo Brazil

**Keywords:** Metabolism, Muscle stem cells, Mitochondria, Bioenergetics

## Abstract

**Background:**

Skeletal muscle stem cells (satellite cells) are well known to participate in regeneration and maintenance of the tissue over time. Studies have shown increases in the number of satellite cells after exercise, but their functional role in endurance training remains unexplored.

**Methods:**

Young adult mice were submitted to endurance exercise training and the function, differentiation, and metabolic characteristics of satellite cells were investigated *in vivo* and *in vitro*.

**Results:**

We found that injured muscles from endurance‐exercised mice display improved regenerative capacity, demonstrated through higher densities of newly formed myofibres compared with controls (evidenced by an increase in embryonic myosin heavy chain expression), as well as lower inflammation (evidenced by quantifying CD68‐marked macrophages), and reduced fibrosis. Enhanced myogenic function was accompanied by an increased fraction of satellite cells expressing self‐renewal markers, while control satellite cells had morphologies suggestive of early differentiation. The beneficial effects of endurance exercise were associated with satellite cell metabolic reprogramming, including reduced mitochondrial respiration (O_2_ consumption) under resting conditions (absence of muscle injury) and increased stemness. During proliferation or activated states (3 days after injury), O_2_ consumption was equal in control and exercised cells, while exercise enhanced myogenic colony formation. Surprisingly, inhibition of mitochondrial O_2_ consumption was sufficient to enhance muscle stem cell self‐renewal characteristics *in vitro*. Moreover, transplanted muscle satellite cells from exercised mice or cells with reduced mitochondrial respiration promoted a significant reduction in inflammation compared with controls.

**Conclusions:**

Our results indicate that endurance exercise promotes self‐renewal and inhibits differentiation in satellite cells, an effect promoted by metabolic reprogramming and respiratory inhibition, which is associated with a more favourable muscular response to injury.

## Introduction

Satellite cells are the resident stem cells of the skeletal muscle, capable of regenerating the tissue throughout adulthood in response to insults.^1^ Regeneration occurs by activation of satellite cells, which can then differentiate and either fuse with existing multinuclear contractile myofibres and repair them or generate new myofibres by fusing with other activated satellite cells.

Satellite cells express paired box protein 7 (Pax7), a critical regulator of muscle stem cell maintenance and activation. Indeed, loss of this protein leads to lack of satellite cell expansion and differentiation in both neonatal and adult muscles.^2^, ^3^ Activation of these stem cells into committed myoblasts occurs because of extrinsic myogenic stimuli and typically involves expression of the myogenic regulatory factor MyoD. The committed myogenic progenitors then actively proliferate to generate sufficient committed myoblasts, as well as produce differentiated myocytes. Activated cells can also return to quiescence and self‐renew, a process marked by Spry1 and Notch expression.^4^, ^5^, ^6^, ^7^, ^8^, ^9^, ^10^, ^11^

One of the most effective stimuli to induce satellite cell activation in adult muscles is exercise. Indeed, resistance or workload exercise has long been known to activate and increase numbers of satellite cells, as well as promote myonuclear expansion and hypertrophy.^12^ Interestingly, however, the effects of aerobic endurance exercise on satellite cell function are less understood, and mixed results have been described in the literature.^3^, ^13^, ^14^ This is an important point of study, because endurance exercise is well known to improve skeletal muscle fitness^15^ and is therefore expected to affect satellite cell activation, self‐renewal, and differentiation.^16^ Increased satellite cell content has consistently been described after endurance training in young^17^, ^18^ and old animals.^19^, ^20^, ^21^ In addition, aerobic exercise conditioning increases the capacity of the skeletal muscle to repair after injury in aged mice.^22^ However, the mechanisms involved in the changes in the function of satellite cells promoted by endurance exercise training have not yet been investigated.

Recent studies have shown that mitochondria may play a role in the regulation of myogenesis. Mitochondrial function in differentiated myocytes is modified by endurance exercise, which promotes increased biogenesis of the organelle, as well as up‐regulated mitochondrial quality control pathways.^23^, ^24^, ^25^ Indeed, respiratory capacity per mitochondrion is lower in middle aged compared with young humans and may be an important factor in age‐related muscle dysfunction and differences in fibre‐type distribution, which are partially prevented by exercise.^24^, ^26^, ^27^, ^28^, ^29^ Mitochondrial function is also involved in stem cell activation and differentiation (reviewed in Khacho and Slack^30^). Thus, understanding how mitochondria are involved in satellite cell function could provide insights into mechanisms that regulate muscle health.

Although the type of mitochondrial functional alteration during commitment to differentiation is variable with stem cell fate,^31^ activation of oxidative phosphorylation is often required upstream of differentiation, while suppression is necessary for self‐renewal in pluripotent stem cells.^30^ Little is known, however, about how changes in mitochondrial bioenergetics are related to satellite cell activation, self‐renewal, or differentiation. In this study, we investigated the effects of endurance exercise training on skeletal muscle regeneration and satellite cell function. Surprisingly, we found that endurance exercise induces a significant repression of satellite cell oxidative phosphorylation, which stimulates self‐renewal.

## Methods

### Animals

Two‐month‐old, young adult and still growing male C57BL/6 mice were purchased from AniLab, Paulínia, Brazil. All experimental procedures were conducted in agreement with Ethical Principles in Animal Research of the Brazilian College of Animal Experimentation (CONCEA) and reviewed and approved by the local animal care and ethics committee (137/2017). Mice were housed 3–4 per cage and maintained on a 12:12 h light–dark cycle in a temperature‐controlled environment (22°C) with *ad libitum* access to standard laboratory chow (Nuvital Nutrientes, Colombo, Brazil) and water.

### Incremental speed test and endurance exercise protocol

Mice were submitted to incremental speed exercise testing on a motor treadmill before and after the experimental period of endurance training. After adaptation to the treadmill environment for 1 week (10 min per session), mice were placed on the treadmill at 0% inclination and allowed to acclimatize for at least 10 min. Incremental speed increases then started at 10 m/min during 10 min and were increased by 3 m/min every minute (*n* = 7, control; *n* = 13, exercised). Mice that could no longer run for over 1 min were considered exhausted and removed from the treadmill. This incremental speed test provided the maximal running duration (min), distance (m), and velocity (m/min).

Endurance exercise was randomly assigned for sedentary (control) and exercised mice as 60% of the maximum intensity (maximal aerobic capacity) reached during the incremental speed test, as previously described.^32^, ^33^, ^34^, ^35^ Exercised mice performed moderate‐intensity running exercises on a motorized treadmill for 5 weeks (from the 8th to 13th weeks of age), 5 days a week, 60 min/day. The running velocity during the period of physical training was gradually increased to 60% of maximal aerobic intensity (described earlier). During 5 weeks, treadmill‐running skills were maintained in sedentary (control) mice for 5 min (10 m/min), twice a week, to avoid any interference of treadmill stress. This activity in control animals did not alter maximal exercise capacity.^34^ Body weights and food consumption were recorded weekly.

Forty‐eight hours after the last exercise at 5 weeks of endurance training, run capacity (maximal running times, velocities, and distances) was evaluated in all mice.^32^, ^33^, ^34^, ^35^, ^36^ After this session, the animals were submitted to live animal experiments, as described in the succeeding text, or anaesthetized with isoflurane and sacrificed by cervical dislocation for tissue or cell collection. Tissues were quickly removed, weighed, immediately frozen in liquid nitrogen, and stored at −80°C.

### Indirect calorimetry, spontaneous physical activity, and maximal oxygen consumption (VO_2_max) determination

A room with restricted access was dedicated for this experiment. Oxymax (Columbus Instruments, Columbus, OH, USA) and TSE Systems were used to monitor real‐time changes in gas concentrations. Animals (*n* = 4, control; *n* = 4, exercised) were placed in individual 30‐cm‐diameter cages. Each mouse was acclimatized to an individual chamber 24 h before the measurements, and data were collected every 20 min for 24 h. Results shown were collected during the active period, corresponding to the early dark hours.

Infrared detection was used to measure locomotor activity in parallel with the indirect calorimetry assessments. Data were recorded for at least one full circadian period (24 h), and results shown were collected during the early dark hours. Movements were added for every minute and detected simultaneously on all three axes: forward and backward (*x*), side to side (*y*), and up and down (*z*).

Coupling of the treadmill with adjustable intensity to indirect calorimetry and an open‐circuit calorimeter allowed for the assessment of maximal oxygen consumption (VO_2_max). Prior to the experiment, a familiarization session was run. Gases were measured every 10 s. Average VO_2_ and running distance were calculated for each mouse (*n* = 10, control; *n* = 12, exercised).

### Muscle lesions

For muscle regeneration studies, injury was induced 24 h after the last endurance exercise session. Mice (*n* = 4, control; *n* = 4, exercised) were anaesthetized by inhalation using isoflurane. The belly region of the tibialis anterior muscle was injected intramuscularly with 50 μL of 1.2% w/v barium chloride. Seven days after the injury, the animals were anaesthetized with isoflurane and sacrificed by cervical dislocation. The muscles were quickly removed and stained.

For satellite cell activation/proliferation studies, muscle injury was induced 24 h after the last exercise session. Mice were anaesthetized, and the belly region of the tibialis anterior, gastrocnemius, quadriceps, and triceps brachii muscles were injected intramuscularly with 10 μL of 1.2% w/v barium chloride. Three days after the injury, the animals were sacrificed, skeletal muscles were quickly removed, and satellite cells were isolated as described in the succeeding text.

### Stains and microscopy

For haematoxylin and eosin (H&E) staining analysis, injured muscles were embedded into tragacanth and Tissue‐Tek® O.C.T. (Sakura Quality‐Somagen, AR, Netherlands) solution and frozen in liquid nitrogen. Sections (10 μm thick) were obtained from the mid‐belly region of the injured tibialis anterior muscle (*n* = 4, control; *n* = 4, exercised) using a Leica CM3050 cryostat (Wetzlar, Germany). Sections were H&E stained and photographed using a Nikon Eclipse E1000 microscope (Nikon's Microscopy, MA, USA) coupled to a DXM 1200 camera. Non‐overlapping images from the central region of each section were analysed using the cell counter plugin for ImageJ software developed at the National Institutes of Health and the Laboratory for Optical and Computational Instrumentation, MD, USA (central nuclei or infiltrating cells per mm^2^). Mean cross‐sectional areas were determined by measuring the circumferences (μm^2^) of 25 adjacent fibres in six sections per slide per animal, thus a total of 150 fibres per animal (*n* = 4, control; *n* = 4, exercised). Data represent the averages of the mean values for each mouse in each group. Analysis was performed blinded.

Picrosirius Red was used to evaluate collagen content in injured muscles, as described by Junqueira *et al*.^37^ Sections were photographed using a polarized light microscope, and images were captured on a JVC TK‐1280E digital color video camera (JVC, Burladingen, GER) coupled to the microscope. A section of muscle fibres surrounded by perimysium, containing 10–100 fibres (*n* = 4, control; *n* = 4, exercised), was imaged as previously reported.^37^ Analysis was performed blinded.

### Dual X‐ray absorptiometry measurements

Forty‐eight hours after the last endurance exercise session, mice (*n* = 4, control; *n* = 4, exercised) were scanned using the FX PRO In‐Vivo Imaging Systems (BRUKER Corporation, MA, USA.). A combination of xylazine (10 mg/kg) and ketamine (90 mg/kg) was used for sedation. Fat area was measured in the abdomen (top of the pelvis to the lowermost rib) and calculated as abdominal fat divided by the total abdominal tissue. Lean area was evaluated in the upper hindlimbs.

### Muscle stem cell isolation, culture, and clonal analysis

Muscle stem cells were isolated from pooled intact extensor digitorum longus, gastrocnemius, quadriceps, soleus, tibialis anterior, and triceps brachii muscles, as previously described.^38^, ^39^, ^40^ Briefly, muscles were removed, minced, mechanically dissociated, and filtered twice with 70 mm strainers. The resulting tissue was centrifuged and digested with 2% type II collagenase, 0.25% trypsin, and 0.1% DNAse. Primary myoblasts were purified by 2–3 pre‐plating steps^41^ and cultured in Dulbecco's modified Eagle's medium containing 1% penicillin and 20% foetal bovine serum. We typically isolated 450 000–550 000 satellite cells from the pooled hindlimb and forelimb muscles of each mouse using this protocol. Cell number and viability were determined using a Neubauer chamber and trypan blue vital staining, respectively. Cells were not passaged. In purity checks, 84.6% of the isolated cells with 4,6‐diamidino‐2‐phenylindole (DAPI)‐stained nuclei were Pax7 positive (*n* = 8), as assessed by immunofluorescence (described in the succeeding text).

For clonal analysis, isolated cells were grown for 168 h in low‐glucose Dulbecco's modified Eagle's medium containing 1% penicillin and 20% foetal bovine serum on Matrigel‐coated six‐well plates (5000 cells per well) and then fixed with 3.8% paraformaldehyde, dried for 24 h, dyed with 1% crystal violet, and washed. After 24 h, clone colonies (clusters of approximately 50 cells) were manually counted using a Nikon SMZ 745 stereoscopic microscope (Nikon Instruments, NY, USA) with 7.5 zoom (*n* = 4, control; *n* = 4, exercised).

### Immunofluorescence

Satellite cells from control and exercised mice were plated for 96 h on Matrigel‐coated 12‐well plates (100 000 cells per well), then fixed with 3.8% paraformaldehyde for 10 min, permeabilized with 0.3% Triton X‐100, and blocked in 1% bovine serum albumin, 0.3% Triton X‐100, and 0.1% sodium azide in phosphate‐buffered saline for 2 h at room temperature. Cells were incubated with primary antibody (Pax7 monoclonal mouse IgG1, Abcam 199010; 1:50, Cambridge, MA, USA) at 4°C overnight followed by DAPI for 1 h and fluorescent‐labelled secondary antibodies (goat anti‐mouse IgG conjugated to Alexa Fluor^®^ 488; 1:1000, Life Technologies Corporation, CA, USA). Images were captured with CoolSNAP HQ CCD camera (Photometrics, Tucson, AZ, USA) driven by IPLab software (Scanalytics Inc., Milwaukee, WI, USA) using a Leica DMI 6000B fluorescent microscope (Mannheim, Germany). Quantification used the cell counter plugin for ImageJ software.

Injured muscle cross sections were fixed with 4% paraformaldehyde at room temperature for 10 min, washed with phosphate‐buffered saline with 0.1% Tween 20 (PBS‐T), and incubated for 1 h with blocking solution (1% bovine serum albumin in PBS‐T). Samples were incubated overnight at 4°C with primary rabbit anti‐eMHC antibody (1:500, Origene Cat#TA349138, Rockville, MD, USA) and anti‐CD68 (1:500, mAb #76437). After washing with 0.1% PBS‐T, the secondary antibody was added in blocking solution for 1 h in the dark, followed by further washing. Slides were mounted with VECTASHIELD for fluorescence with DAPI (Cat# H‐1200, Vector Labs, CA, USA). Images were captured with a CoolSNAP HQ CCD camera (Photometrics) driven by IPLab software (Scanalytics Inc.) using a Leica DMI 6000B fluorescence microscope (Mannheim, Germany). Quantification was conducted using the cell counter plugin for ImageJ software.

### Morphological analysis

Satellite cells from control and exercised mice were cultured for 96 h on Matrigel‐coated 12 well plates (100 000 cells per well, *n* = 4, control; *n* = 4, exercised). Images were acquired on a Nikon Eclipse TS100 microscope, and quantification of spindle‐like and elongated morphology was conducted using the cell counter plugin for ImageJ.

### Metabolic flux analysis

Oxygen consumption rates (OCRs) and extracellular acidification rates (ECARs) of muscle stem cells were measured using a Seahorse XF24 Analyzer (Seahorse Bioscience, MA, USA), as described previously.^31^, ^42^ Cells (animal *n* = 4, control; *n* = 4, exercised) were seeded on Matrigel‐coated XF24 24‐well microplates at 20 000 cells per well for 120 h. Before the assay, the media were removed and replaced by 500 mL of low‐glucose (5 mM) assay medium, without sodium bicarbonate, to allow for ECAR measurements. ATP synthesis‐linked O_2_ consumption and proton leak‐driven respiration were determined by the addition of oligomycin (1 μg/mL). The uncoupler 2,4‐dinitrophenol (2,4‐DNP, 200 μM) was added to promote maximal respiratory capacity. All respiratory modulators were used at concentrations determined through preliminary titration analyses.^31^, ^42^ Rotenone (1 μM) and 1 μg/mL antimycin A were added to ablate mitochondrial O_2_ consumption.

### RNA extraction and real‐time polymerase chain reaction analysis

Cells were cultured on six‐well plates (250 000 cells per well) for 120 h (animals used were *n* = 8, control, and *n* = 8, exercised). For mtDNA quantifications, DNA samples were prepared using a DNeasy^®^ Blood & Tissue Kit (Qiagen, SP, Brazil). Real‐time quantitative polymerase chain reaction (qPCR) was performed using SYBR Green PCR Master Mix (Applied Biosystems, MA, USA). The following genes were analysed: hypoxanthine‐guanine phosphoribosyltransferase (HPRT) for nDNA and mitochondrially encoded NADH:ubiquinone oxidoreductase core subunit 1 (MT‐ND1) for mtDNA copy number. Fold changes were calculated by the 2^−ΔΔCT^ method. The primer sequences are presented in Supporting Information, *Table* *S1*.

Total RNA was purified using TRIzol reagent (Invitrogen Life Technologies, Rockville, MD, USA) and quality checked using 260/230 and 260/280 nm scores. Equivalent contents of RNA were reverse transcribed using a SuperScript III cDNA Synthesis Kit (Invitrogen, CA, USA). The cDNA synthesized was stored at −20°C prior to the real‐time PCR assay. Amplification was performed using Platinum^®^ SYBR^®^ Green qPCR SuperMix UDG (Invitrogen Life Technologies, Carlsbad, CA, USA) and evaluated by real‐time PCR using the Rotor Gene 3000 apparatus (Corbett Research, Mortlake, Australia). Primer sequences were designed using information contained in the Gene Bank of the National Center for Biotechnology Information. Gene expression was quantified using qBase software (Qbase, WA, UK), as described previously.^43^ Expression was normalized using the internal control gene HPRT. Fold changes relative to HPRT were calculated by the 2^−ΔΔCT^ method. Primers were selected using PrimerBank (https://www.exxtend.com.br); sequences are presented in *Table* *S1*.

### Muscle stem cell transplantation

The tibialis anterior muscles of the hosts, 14‐week‐old male mice, were prepared by BaCl_2_ injury as described earlier 1 day before transplantation; then, 20 000 freshly isolated muscle stem cells from control or exercised mice in 30 μL media were slowly injected into the mid‐belly of the muscle of a recipient anaesthetized host (control) mouse (*n* = 4, control; *n* = 4, exercised; and *n* = 4, antimycin A). Seven days after transplantation, the muscles were quickly removed for microscopy.

### Statistical analysis

Results are presented as mean ± standard error of the mean and were analysed using GraphPad Prism Software 4.0 (GraphPad Company, CA, USA). Data were compared using *t*‐tests to determine if the means of two sets of data are significantly different from each other or one‐way analysis of variance used to compare means of two or more samples, followed by the Bonferroni *post hoc* test. Asterisks denote statistically significant differences vs. control. Grubb's test was used to exclude outliers.

## Results

### Endurance exercise increases performance and promotes a shift in substrate use

**Figure 1:**
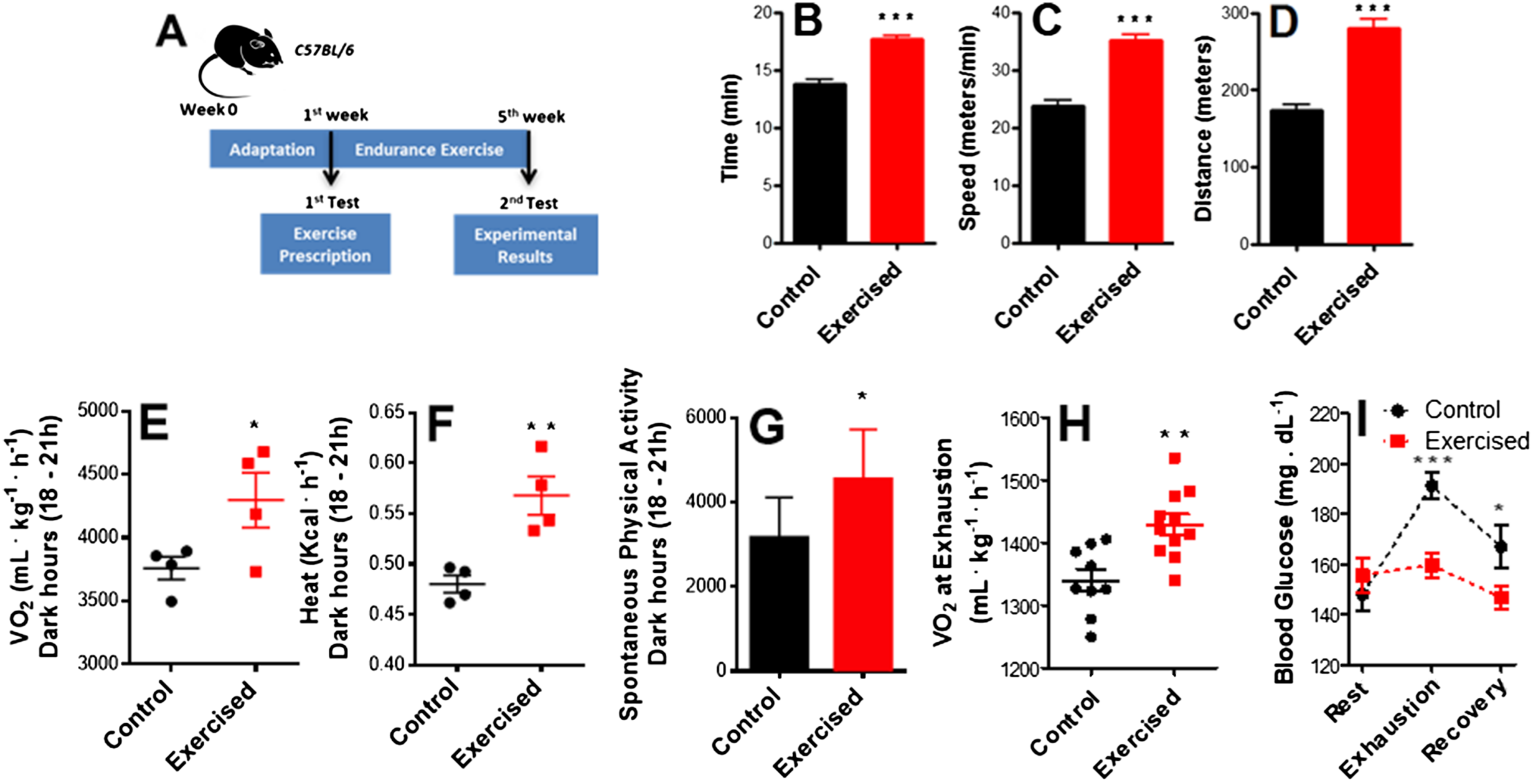
Endurance exercise induces a performance‐dependent shift and changes energy substrate usage. (A) Schematic panel. (B) Running time. (C) Running speed. (D) Distance covered in the run‐to‐exhaustion test (*n* = 7, control; *n* = 13, exercised). (E) O_2_ consumption during the dark hours (18–21 h) measured by indirect calorimetry. (F) Heat production during the dark hours measured by indirect calorimetry. (G) Spontaneous physical activity during the dark hours (*n* = 4, control; *n* = 4, exercised). (H) O_2_ consumption at exhaustion (*n* = 9, control; *n* = 11, exercised). (I) Resting blood glucose, blood glucose during run‐to‐exhaustion, and blood glucose at 10 min rest after exhaustion (*n* = 7, control; *n* = 13, exercised). Asterisks denote statistically significant differences (^*^ *P* < 0.05, ^**^ *P* < 0.01, and ^***^ *P* < 0.001) vs. control.

Mice were submitted to an endurance exercise protocol for 5 weeks (*Figure* 1A), a time span sufficient to observe many of metabolic phenotypes associated with exercise.^32^, ^33^, ^34^, ^35^ Their performance was then evaluated 48 h after the last exercise training session. Consistent with prior reports, exercised mice presented an increase in running time (*Figure* 1B), speed (*Figure* 1C), and distance (*Figure* 1D) in the run‐to‐exhaustion test. Exercised mice also displayed a shift in energy substrate usage at rest analysed through indirect calorimetry, as evidenced by increased O_2_ consumption (*Figure* 1E) and heat production (*Figure* 1F). Interestingly, spontaneous physical activity (*Figure* 1G) was also increased in exercised mice.^44^ Performance was then measured under run‐to‐exhaustion conditions. Oxygen consumption (*Figure* 1H) was again higher in exercised animals. Blood glucose monitoring during the run‐to‐exhaustion test showed more steady blood glucose levels in exercised animals (*Figure* 1I).^34^, ^45^ Overall, these data indicate that the exercise regimen promoted enhanced performance associated with metabolic modifications.

**Figure 2:**
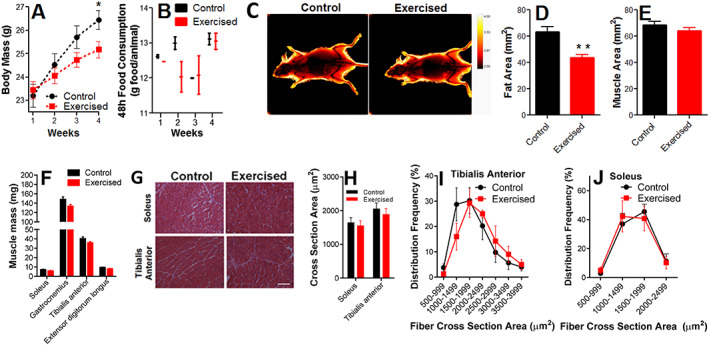
Body fat content is decreased by endurance exercise, while muscle content is preserved. (A) Body mass changes. (B) Food consumption (*n* = 4, control; *n* = 4, exercised). (C) Dual X‐ray absorptiometry scans, representative image. (D) Fat area. (E) Muscle area (*n* = 6, control; *n* = 6, exercised). (F) Muscle mass (*n* = 7, control; *n* = 9, exercised). (G) Representative images of cross‐sectional areas. (H) Cross‐sectional area quantifications. (I and J) Frequency distribution of the cross‐sectional areas of the fibres (*n* = 4, control; *n* = 4, exercised). Scale bar = 100 μm. Asterisks denote statistically significant differences (^*^ *P* < 0.05; ^**^ *P* < 0.01) vs. control.

Endurance exercise resulted in body mass reduction (*Figure* 2A) that was not the sole result of changes in food consumption (*Figure* 2B). Dual X‐ray absorptiometry scans were used to measure body composition (*Figure* 2C, representative images) and revealed a significant decrease in the relative area occupied by adipose tissue in this exercised group (*Figure* 2D), with no difference in lean body composition (*Figure* 2E). Indeed, muscle weights (*Figure* 2F) did not change nor did averages or the distribution of cross‐sectional areas of the soleus muscle (in which oxidative fibres are predominant) or tibialis anterior muscle (mostly glycolytic; *Figure* 2G–2J). Other organs such as the heart and liver also did not present altered mass (results not shown), suggesting that weight loss is specific to fat depots.

### Endurance exercise enhances skeletal muscle repair

**Figure 3:**
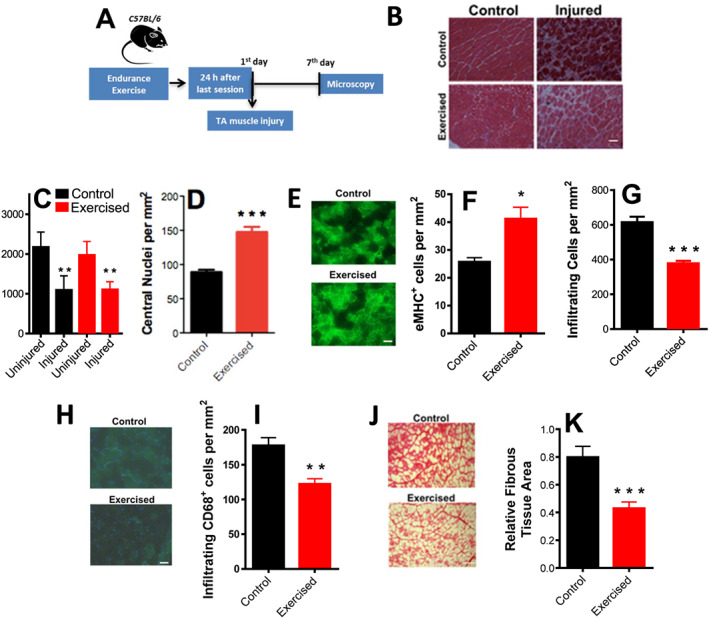
Aerobic fitness enhances skeletal muscle repair and promotes anti‐inflammatory and anti‐fibrotic effects. (A) Schematic panel. (B) Illustrative images of fibre cross sections (scale bar = 100 μm). (C) Cross‐sectional area quantifications of control and exercised samples with and without injury. (D) Centrally nucleated (newly formed) myofibre counts in injured muscles. (E) Representative images of eMHC^+^‐stained fibres. (F) Quantification of eMHC^+^ fibres. (G) Infiltrating inflammatory cell counts in injured muscles. (H) Representative low magnification images, CD68^+^ (macrophage) staining in green and DAPI in blue. (I) Quantification of CD68^+^ cells (macrophages). (J) Picrosirius Red‐stained injured muscles, representative images. (K) Relative fibrous area quantification (*n* = 4, control; *n* = 4, exercised). Scale bars = 50 μm (E and H) and 100 μm (B and J). Asterisks denote statistically significant differences (^*^ *P* < 0.05, ^**^ *P* < 0.01, and ^***^ *P* < 0.001) vs. controls.

Endurance exercise has been shown to protect against loss of satellite cells during ageing, as well as increase their number and capacity to differentiate.^17^, ^19^, ^46^ We thus verified if satellite cell‐mediated muscle regeneration after injury (*Figure* 3A) was affected by endurance exercise. H&E microscopies of uninjured and injured tibialis anterior muscles from control (sedentary) or exercised animals are shown in *Figure* 3B and uncover no changes promoted by exercise in the decreased average cross‐sectional area measurements with injury (*Figure* 3C). However, injured muscles from exercised mice had higher density of fibres with central nuclei, suggestive of newly formed myofibres due to improved regenerative capacity (*Figure* 3D). Quantification of embryonic myosin heavy chain (eMHC)‐expressing myofibres, a hallmark of regeneration in adult muscle, was also increased (*Figure* 3E and 3F). Endurance exercise training also decreased the extent of inflammatory cell infiltration (*Figure* 3G) and the presence of cells expressing the macrophage marker CD68 (*Figure* 3H and 3I). Indeed, Picrosirius Red staining (*Figure* 3J), used to quantify the fibrous tissue area (*Figure* 3K), demonstrates that exercised animals showed lower collagen content. Overall, these results show that endurance exercise training significantly accelerates the regeneration efficiency of muscle and improves muscle regenerative response, resulting in higher new myofibre appearance, lower inflammation, and prevention of fibrosis.

### Endurance exercise enhances satellite cell self‐renewal

**Figure 4:**
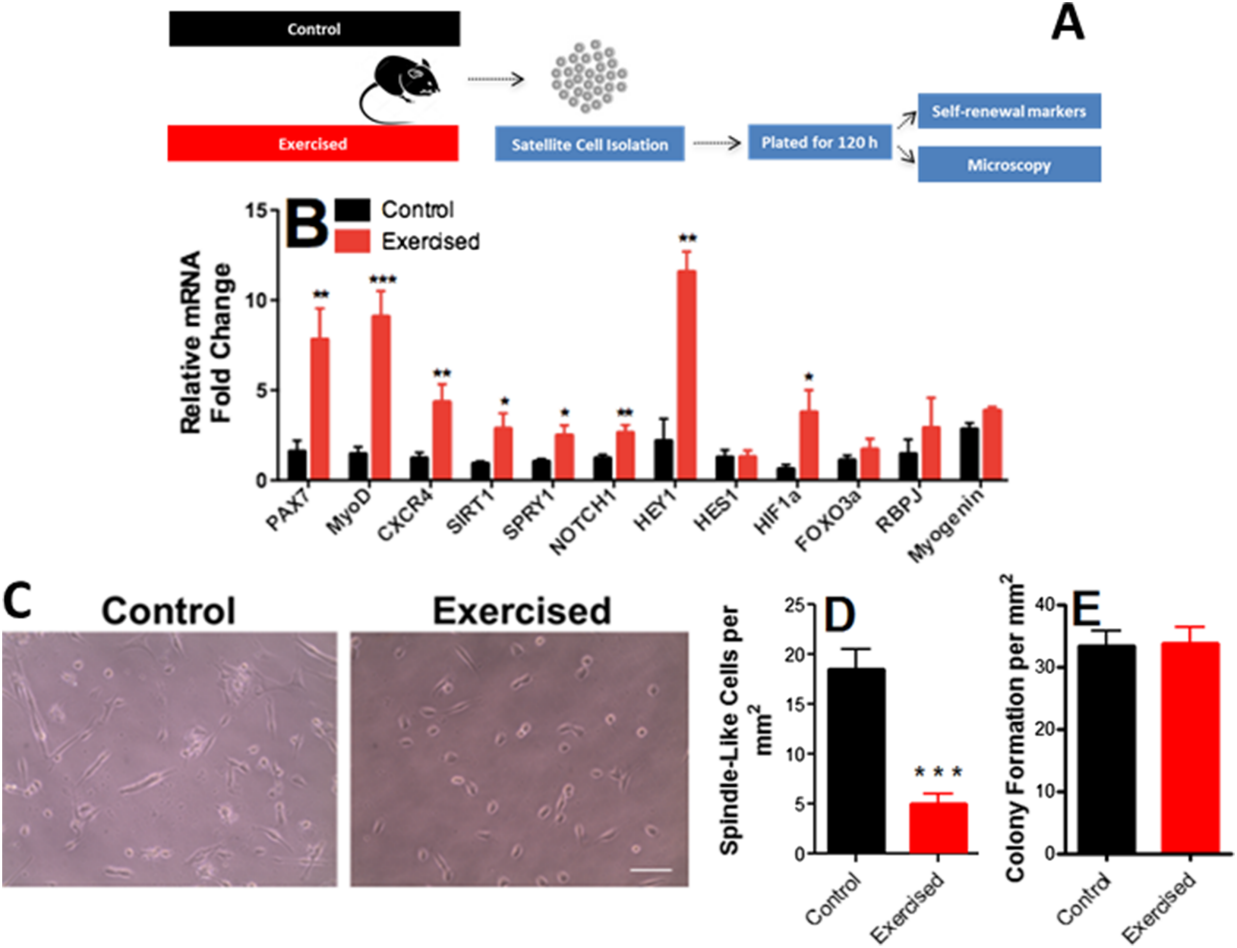
Endurance exercise enhances self‐renewal and quiescence markers in satellite cells. (A) Schematic panel. (B) Expression of Pax7 (a satellite cell‐specific marker that increases upon activation), MyoD1 (an activation marker), CXCR4 (a satellite cell marker), Sirt1 (a self‐renewal marker), Spry1 (a return‐to‐quiescence marker), Notch1, Hey1, Hes1, and HIF1α (self‐renewal markers), FOXO3a (quiescence marker), Rbpj (involved in Notch1 signalling), and myogenin (a differentiation marker; *n* = 8, control; *n* = 8, exercised), relative to HPRT. (C) Representative images of satellite morphology. (D) Quantification of elongated, spindle‐like (more differentiated) cells. (E) Myogenic colony formation (*n* = 4, control; *n* = 4, exercised). Asterisks denote statistically significant differences (^*^ *P* < 0.05, ^**^ *P* < 0.01, and ^***^ *P* < 0.001) vs. control.

The higher density of newly formed fibres and lower inflammation in endurance‐exercised mice suggest changes in satellite cell population.^19^, ^46^ We thus isolated and plated satellite cells from exercised and control animals (*Figure* 4A). Cell preparations consistently presented high purity, as indicated by immunofluorescence assays for the satellite cell marker Pax7 (see Methods, ^47^). After 120 h of growth in culture without passaging, satellite cells were proliferative and at 70–80% confluence. At this time point, the expression of Pax7, MyoD1 (a marker for satellite cell activation), and CXCR4 (a satellite cell membrane marker) were significantly enhanced in cells isolated from exercised animals (*Figure* 4B). Self‐renewal markers Sirt1 (which regulates metabolic state and proliferation^48^, ^49^) and HIF1α^50^ were also increased by exercise, as were Spry1, Notch1, and Hey1, which signal myoblast return to quiescence, maintaining the satellite cell pool and long‐term muscle integrity.^48^ FOXO3a expression (a quiescence marker) and myogenin (a differentiation marker) were not modified (*Figure* 4B). Overall, this expression profile suggests endurance exercise, promotes activation, but prevents final satellite cell differentiation, maintaining self‐renewal or return‐to‐quiescence phenotypes.

To functionally assess the differentiation of these cells, cell morphology was evaluated (*Figure* 4C and 4D). Control satellite cells from sedentary animals showed spindle‐like elongated morphology, suggestive of early differentiation and loss of stemness, while endurance exercise promoted a more undifferentiated morphology. Despite changes in differentiation markers and morphology, no difference in colony formation ability was observed between exercised and control cells (*Figure* 4E). Overall, our results suggest that endurance exercise promotes satellite cell self‐renewal and quiescence, but inhibits differentiation, without affecting proliferation.

### Endurance exercise reduces satellite cell O_2_ consumption rates under physiological, but not activated states

**Figure 5:**
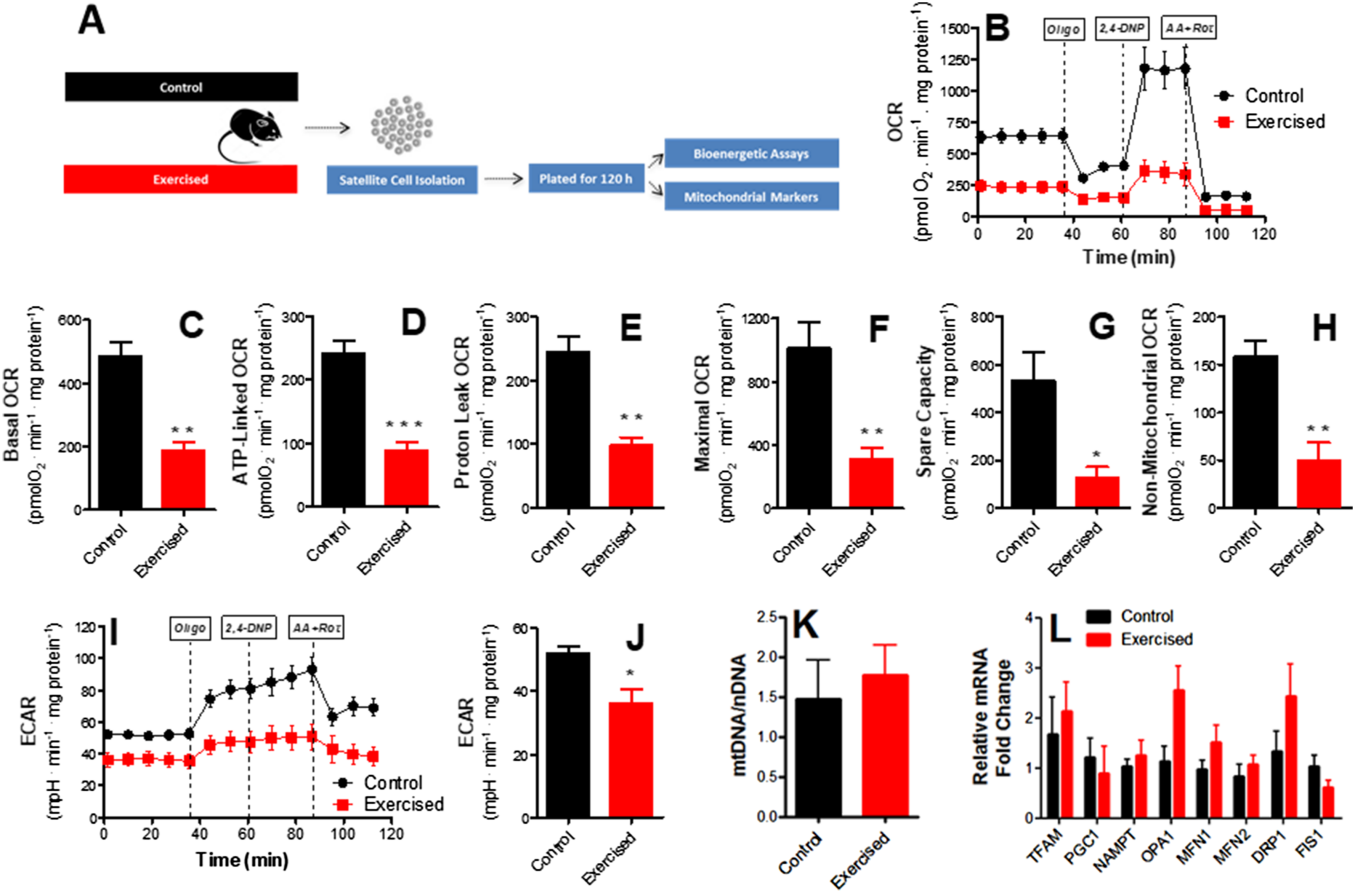
Endurance exercise reduces mitochondrial O_2_ consumption in skeletal muscle stem cells. (A) Schematic panel. (B) Typical traces of real‐time O_2_ consumption rate (OCR) measurements in control and exercised cells determined using the Seahorse XF Analyzer. Oligomycin, 2,4‐DNP, and rotenone plus antimycin A were added where indicated. (C) Basal O_2_ consumption rates. (D) ATP production‐dependent OCR (basal minus oligomycin‐insensitive). (E) Proton leak (oligomycin‐insensitive) OCR. (F) Maximal OCR in the presence of 2,4‐DNP. (G) Spare respiratory capacity (maximal minus basal). (H) Non‐mitochondrial (antimycin plus rotenone‐insensitive) OCR. (I) Typical extracellular acidification rate (ECAR) measurement. (J) ECAR quantification (*n* = 4, control; *n* = 4, exercised). (K) Mitochondrial DNA/nuclear DNA ratio (mtDNA/nDNA) (*n* = 6, control; *n* = 4, exercised). (L) Expression profile of mitochondrial markers (*n* = 8, control; *n* = 8, exercised). Asterisks denote statistically significant differences ^(*^ *P* < 0.05, ^**^ *P* < 0.01, and ^***^ *P* < 0.001) vs. control.

There is literature evidence that satellite cell cycle regulation involves changes in energy metabolism, with modulation of substrate use.^48^, ^51^, ^52^, ^53^ However, mitochondrial oxidative phosphorylation, which is a key regulatory factor in stem cell differentiation,^30^, ^31^ has not been directly assessed in the context of satellite cell modulation. We used a Seahorse Extracellular Flux analyzing system to assess mitochondrial bioenergetics in intact satellite cells isolated from control or endurance‐exercised animals (*Figure* 5A).

A typical trace of real‐time OCR measurements is shown in *Figure* 5B. Endurance exercise promoted overt changes in mitochondrial bioenergetics, with a substantial suppression of basal (physiological) O_2_ consumption rates (the initial part of the trace in *Figure* 5B, quantified in *Figure* 5C). This was in part due to lower ATP‐linked oxygen consumption in exercised cells, or the OCR inhibition promoted by the addition of the ATP synthase inhibitor oligomycin (*Figure* 5D). Additionally, oligomycin‐insensitive O_2_ consumption (*Figure* 5E), which represents the proton leak of the inner membrane, was decreased by exercise. Exercised cells also presented lower maximal respiratory rates, prompted by the addition of the uncoupler 2,4‐DNP (*Figure* 5F) and lower reserve capacity, or the difference between basal and maximal respiration (*Figure* 5G). However, reserve capacity was still present, indicating that basal respiratory levels were not limited by overall respiratory capacity. Finally, exercised cells presented less non‐mitochondrial respiration, measured in the presence of antimycin plus rotenone (*Figure* 5H), although these OCRs were low compared with overall oxygen consumption. Non‐mitochondrial OCRs were subtracted from all other quantifications.

Inhibition of mitochondrial respiration is often accompanied by a shift towards glucose fermentation to lactate. Indeed, when more quiescent, many cells are more fermentative.^54^ However, ECARs (*Figure* 5I and 5J) in exercised satellite cells were significantly decreased, suggesting that they indeed have lower ATP production, and not a shift towards lactate formation. Lower ATP demands in the exercised cells are compatible with the expression profile observed previously, suggestive of a higher return to quiescence induced by endurance exercise.

Despite the changes in satellite cell respiratory capacity, exercise did not alter mitochondrial DNA levels relative to nuclear DNA (*Figure* 5K). This indicates that the changes in respiration are not accompanied by changes in mitochondrial mass. Indeed, the expression of both proteins involved in mitochondrial biogenesis and morphology was not significantly altered by exercise (*Figure* 5L). Overall, our results show that, despite a lack of modulation of mitochondrial mass, endurance exercise induces a repression of mitochondrial respiration in satellite cells, as well as lower energy demands.

**Figure 6:**
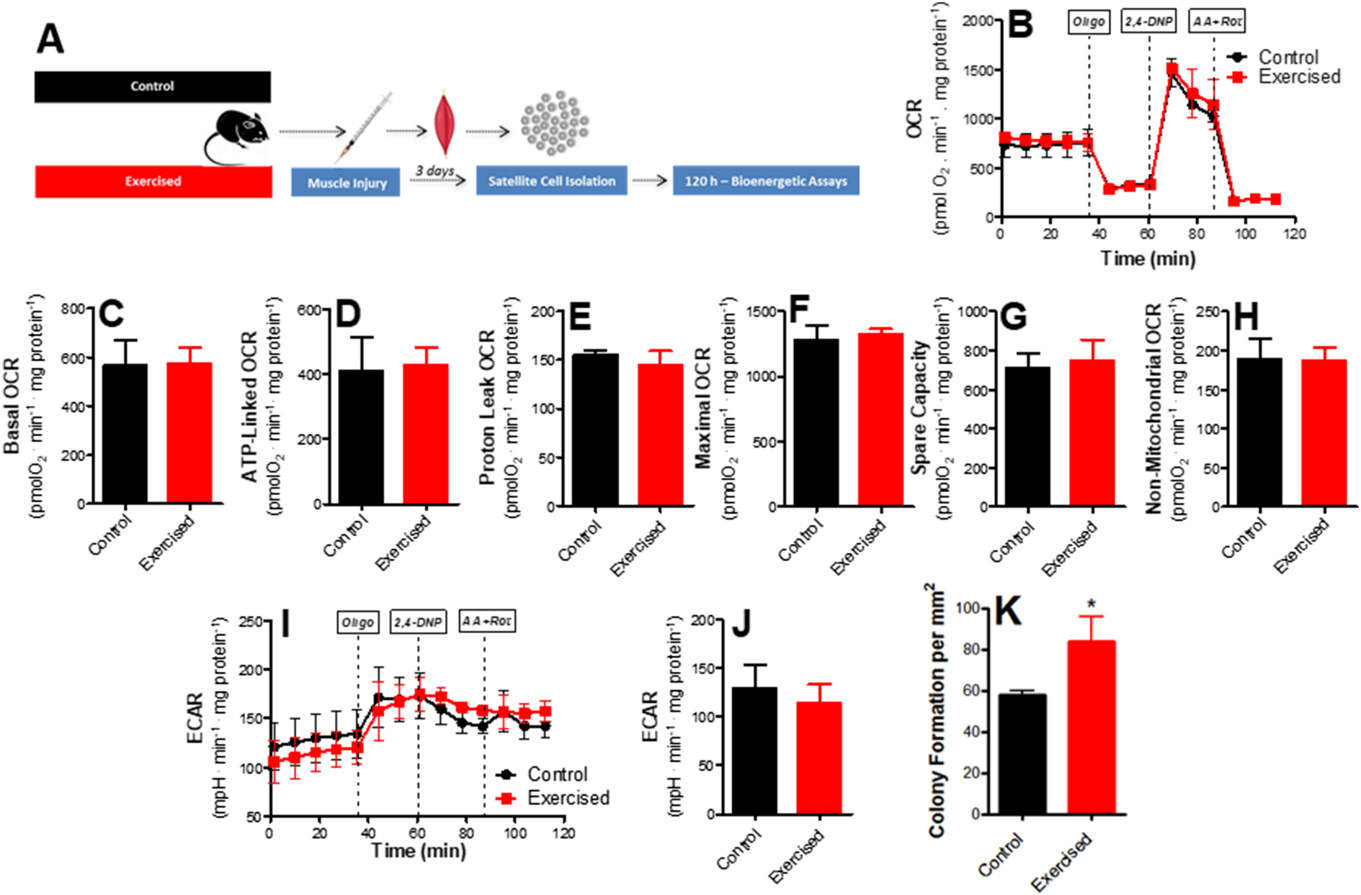
Mitochondrial O_2_ consumption is unaffected in muscle stem cells from injured animals. (A) Schematic panel. (B) Typical trace. Oligomycin, 2,4‐DNP, and rotenone plus antimycin A were added where indicated. (C) Basal O_2_ consumption rates. (D) ATP production‐dependent oxygen consumption rate (OCR). (E) Proton leak OCR. (F) Maximal OCR. (G) Spare respiratory capacity. (H) Non‐mitochondrial OCR. (I) Typical extracellular acidification rate (ECAR) trace. (J) ECAR quantification (*n* = 3, control; *n* = 3, exercised). (K) Myogenic colony formation. Asterisks denote statistically significant differences (^*^ *P* < 0.05) vs. control.

Mitochondrial OCRs were found to be decreased by endurance exercise, an effect associated with a more quiescent expression profile. However, oxidative metabolism is important for stem cell proliferation and activation.^30^, ^54^ Consequently, we evaluated satellite cell metabolism in control or exercised animals after activation induced by injury (*Figure* 6A). Interestingly, while control OCR values were not significantly altered by injury (compare *Figures* 5 and 6B–6H), under injured conditions, exercised satellite cell OCRs were higher and equal to those observed in non‐exercised cells, under all respiratory states (*Figure* 6B–6H). ECAR values were also equal between control and exercised‐activated satellite cells (*Figure* 6I and 6J). This demonstrates that the quiescent metabolic state of exercised cells can promptly respond to activating stimuli, with enhanced metabolic activity, as reflected by enhanced mitochondrial ATP production and respiratory capacity. Interestingly, although metabolic activity was equal, the exercised cells showed higher colony formation (*Figure* 6K), demonstrating that their activated state is more efficient in energy use towards proliferation.

### Inhibition of mitochondrial O_2_ consumption increases satellite cell self‐renewal and quiescence, while decreasing inflammation upon engraftment

**Figure 7:**
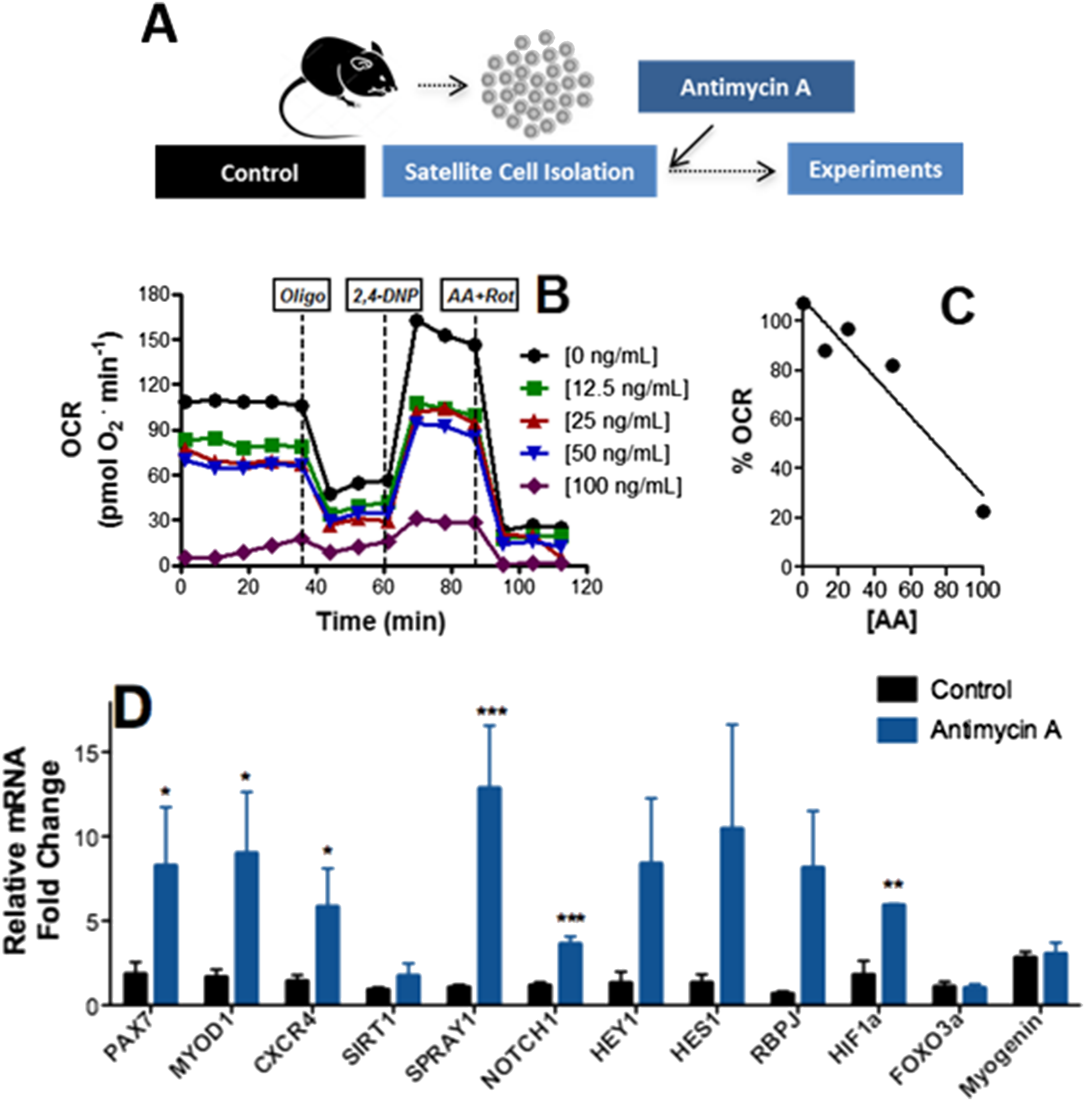
Inhibition of mitochondrial O_2_ consumption promotes satellite cell self‐renewal. (A) Schematic panel. (B) Typical oxygen consumption rate (OCR) trace in the presence of different concentrations of antimycin A. (B and C) Respiratory inhibition vs. antimycin A concentrations. (D) Satellite cell expression profile after 8 h in the presence of 70 ng/mL antimycin A (*n* = 8, control; *n* = 4, antimycin A), relative to HPRT. Asterisks denote statistically significant differences (^*^ *P* < 0.05, ^**^ *P* < 0.01, and ^***^ *P* < 0.001) vs. control.

Because endurance exercise promotes mitochondrial respiratory inhibition associated with enhanced return‐to‐quiescence and self‐renewal markers, we verified if the respiratory inhibition observed was a cause of the changes in expression profile. To do so, we isolated satellite cells from control animals (*Figure* 7A), which have high OCRs, and titrated low concentrations of the specific mitochondrial respiratory inhibitor antimycin A, to achieve a respiratory inhibition similar to that observed in exercised animals (*Figure* 7B and 7C). Our titrations showed that 70 ng/mL antimycin A promoted respiratory inhibition similar to that observed in exercised cells. Interestingly, when we promoted this respiratory inhibition in control cells, the expression profile of non‐exercised cells became very similar to that observed in exercised cells, with an increase in stemness, self‐renewal, and return‐to‐quiescence markers (*Figure* 7D). This indicates that respiratory suppression is sufficient to promote a phenotype more similar to that of endurance‐exercised animals *in vitro*.

**Figure 8:**
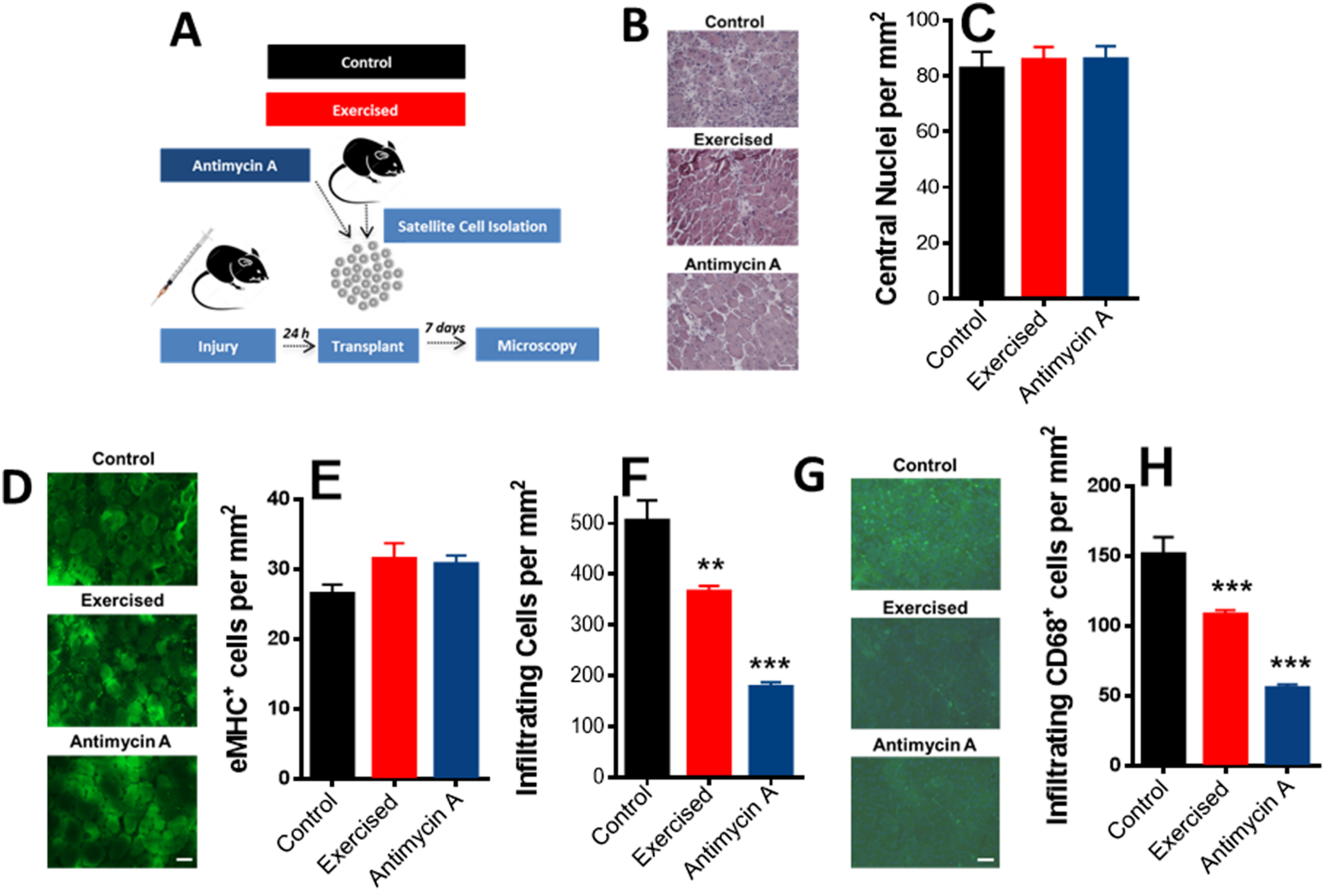
Transplant of exercised or respiratory‐inhibited satellite cells prevents inflammation. (A) Schematic panel. (B) Representative images of transplanted fibre cross sections (scale bar = 100 μm). (C) Centrally nucleated (newly formed) myofibre quantification. (D) Representative images of eMHC^+^‐stained fibres. (E) Quantification of eMHC^+^ fibres. (F) Inflammatory cell quantification. (G) Representative low magnification images, CD68^+^ (macrophage) staining in green and DAPI in blue. (H) Quantification of CD68^+^ cells (macrophages; *n* = 4, control; *n* = 4, exercised; and *n* = 4, antimycin A). Scale bars = 100 μm (B) and 50 μm (D and G). Asterisks denote statistically significant differences (^**^ *P* < 0.01; ^***^ *P* < 0.001) vs. control animals.

Next, we examined if exercised or respiratory‐inhibited satellite cells can lead to changes in responses when transplanted. To do so, isolated cells from control animals treated or not with 70 ng/mL antimycin A, or cells from exercised animals, were transplanted into control animals that had been previously injured to induce engraftment (*Figure* 8A). Illustrative images of tibialis anterior muscles transplanted with control, exercised, and antimycin‐treated satellite cells are shown in *Figure* 8B. While no overt changes in engraftment were noted between treatment groups, as verified by lack of significant changes in centralized nuclei and eMHC^+^ myofibres (*Figure* 8C–8E), both exercised and antimycin‐treated cells lead to muscles with significantly lower inflammation, as assessed by cell infiltration (*Figure* 8F) and cells positive for macrophage marker CD68 (*Figure* 8G and 8H). This anti‐inflammatory effect is most probably related to the enhanced stemness of exercised and respiratory‐inhibited satellite cells.

## Discussion

Endurance exercise is an often practised form of exercise that significantly increases skeletal muscle energy and oxygen demands, thus changing metabolic regulation in the whole body.^55^, ^56^ Indeed, it is a well‐established intervention to preserve muscle function and prevent changes in energy metabolism, such as insulin resistance, that occur with ageing.^57^ Despite its widespread use, the cellular and molecular mechanisms involved in metabolic changes promoted by endurance exercise still remain to be fully understood.

Using a mouse model of endurance exercise involving 5 weeks of daily treadmill runs, we found, predictably, that endurance exercise increases physical performance (*Figure* 1). This occurred in parallel with changes in oxygen consumption at rest and when exercised, as well as changes in exhaustion and recovery glucose levels, confirming exercise induces a shift in whole body metabolism. Indeed, exercised animals were leaner, as indicated by lower fat areas and body mass (*Figure* 2). Perhaps counterintuitively, endurance exercise did not change muscle areas or fibre cross‐sectional areas. These results are in agreement with early work on metabolic effects of exercise, which show that endurance does not promote muscular hypertrophy but leads to enhanced respiratory and lipid oxidation capacity, thus sparing the body from carbohydrate depletion.^58^, ^59^ These early results indicating glucose conservation is a mediator of enhanced stamina in endurance exercise have been confirmed using modern flux analysis techniques.^60^ Recently, peroxisome proliferator‐activated receptor‐δ was found to be a key mediator in the metabolic shift promoted by endurance exercise, increasing palmitate oxidation without an overall change in mitochondrial biogenesis in myocytes.^45^

Although no muscular hypertrophy was observed in exercised animals, prior studies have demonstrated that aerobic exercise changes satellite cell numbers and function, thus improving regenerative response and preserving muscle mass during ageing.^17^, ^18^, ^19^, ^21^, ^22^, ^46^ Our work thus grows upon the findings of Joanisse *et al*.,^22^ who established that aerobic exercise rescues impaired regeneration during ageing. We now show that this change involves modulation of satellite cell metabolism and self‐renewal.

We found that injured muscles in endurance‐exercised animals have improved regenerative efficiency, with accelerated muscle repair measured 7 days after injury, as indicated by a higher number of centrally located nuclei and regenerating myofibres expressing eMHC (*Figure* 3). Notably, endurance exercise training also promoted lower counts of infiltrating cells and cells positive for the macrophage marker CD68, as well as less fibrosis (*Figure* 3). We should note that our experiments were conducted at specific time points and therefore cannot rule out a change in the time course, but not extent, of the final repair. However, this improved regenerative efficiency or speed may be seminal towards skeletal muscle maintenance in ageing.^19^, ^46^, ^61^, ^62^

To uncover changes in satellite cells associated with the increased regenerative response capacity observed in endurance exercise, we isolated these cells using the pre‐plating method.^41^ Our isolation protocol comprises the mechanical and enzymatic dissociation of cells, followed by checks, which indicated that cells obtained were consistently highly pure (see Methods). We found that endurance exercise promoted an increase in return‐to‐quiescence and self‐renewal markers, as well as more undifferentiated morphology, but no changes in colony‐forming capacity (*Figure* 4). Self‐renewal and return to quiescence are pivotal towards maintenance of the satellite cell pool (reviewed in Feige *et al*. and Forcina *et al*.^63^, ^64^) and may explain why endurance exercise promotes more favourable repair after injury.

Interestingly, changes in energy metabolism, and particularly mitochondrial function, have been shown to be central regulatory points in the cell cycle. In many cell types, less differentiation is accompanied by low aerobic metabolism and high glycolytic rates^65^ (reviewed in Xu *et al*.^66^). However, stem cell activation and differentiation is by no means a homogeneous process, and some final cell fates involve decrease of mitochondrial respiratory activity upon commitment to differentiation.^31^ Furthermore, many studies suggesting a shift from fermentation to oxidative metabolism during differentiation are based on gene expression patterns, while energy metabolism is mostly regulated post‐transcription, so gene expression patterns may differ substantially from functional assays.^67^

As a result, we measured metabolic fluxes in satellite cells isolated from control or endurance‐exercised mice. Our results show a clear repression of oxidative metabolism in exercised cells, which present lower mitochondrial oxygen consumption rates under all respiratory states (*Figure* 5), despite a lack of changes in markers of mitochondrial mass, biogenesis, and dynamics. Myogenin and colony formation were not changed under these conditions (*Figure* 4), indicating that proliferation was not responsible for mitochondrial changes. The lower oxygen consumption rates are also not attributable to higher rates of fermentation to lactate, as indicated by lower extracellular acidification rates, and are instead related to lower ATP demands. Indeed, respiratory rates in satellite cells isolated from injured muscles were equal in control and exercised cells (*Figure* 6), showing that exercise did not ablate the ability to respond to injury. Overall, these results suggest that exercise preserves a more quiescent state in unactivated satellite cells, without compromising their activation response. Higher self‐renewal and quiescent capacity may be important for responses towards injury, as indicated by the fact that more newly formed fibres were observed in injured exercised animals (*Figure* 3), and colony formation was more pronounced in satellite cells isolated from exercised animals after injury (*Figure* 6).

Previous studies have analysed the role of oxidative mitochondrial metabolism in satellite cells. Zhang *et al*.^53^ found that aged satellite cells had lower oxidative phosphorylation. However, this was not associated with enhanced quiescence, but instead senescence, as is perhaps expected in ageing. Indeed, the loss of satellite cell function with ageing could be reversed by recovering NAD levels, in a Sirt1‐dependent manner. Consistently, Sirt1 ablation leads to premature differentiation,^48^ a result compatible with our finding that endurance exercise enhances Sirt1 expression, as well as prevents differentiation and promotes self‐renewal. Interestingly, caloric restriction, an intervention that leads to enhanced Sirt1 expression in satellite cells,^51^ does not promote other alterations seen in our exercised satellite cells (depressed respiratory rates in the absence of increased mitochondrial DNA), but instead seems to increase oxidative metabolism and enhance mitochondrial biogenesis. This suggests that satellite cell remodelling by exercise and dietary restriction differ mechanistically, although both interventions help preserve muscle mass during ageing.

Because we observed that exercised satellite cells had lower respiration and higher self‐renewal, we questioned if the return‐to‐quiescence characteristics induced by exercise were a consequence of the lower metabolic rates. Indeed, suppression of oxidative phosphorylation often promotes self‐renewal in stem cells (reviewed in Khacho and Slack^30^). To investigate this, we artificially promoted respiratory inhibition in control satellite cells to similar levels of that observed in exercised cells, by adding low quantities of the classic respiratory inhibitor antimycin A. Remarkably, respiratory suppression alone was sufficient to induce an expression panel in satellite cells similar to that promoted by exercise, with enhanced quiescence and self‐renewal markers (*Figure* 7).

We tested next if satellite cells modified by exercise or respiratory modulation could exhibit changes in engraftment after transplant such as those seen in calorically restricted satellite cells.^51^ We should note that our model has the caveat of not allowing the distinction of host vs. donor cells. We found that neither exercised nor respiratory‐inhibited satellite cells significantly increased centrally localized nuclei or eMHC^+^ fibres in regenerating myofibres when transplanted into control animals after injury (*Figure* 8). This suggests that the regenerative potential of exercised cells observed *in vivo* (*Figure* 3) may depend on characteristics of not only the cells but also their particular niche and thus are not reproduced upon transplantation. However, consistently with their enhanced quiescence markers, both exercised and respiratory‐inhibited satellite cell transplants significantly decreased numbers of infiltrating cells and cells expressing the macrophage marker CD68 (*Figure* 8). This demonstrates that at least part of the beneficial effects of exercise on muscle satellite cell responses can be mimicked by partially suppressing mitochondrial oxidative phosphorylation.

Overall, we demonstrate here that endurance exercise promotes changes in satellite cell function, stemness, self‐renewal, and differentiation. The changes are associated with repression of mitochondrial oxygen consumption. Surprisingly, artificial suppression of respiration in satellite cells mirrors the characteristics of exercise. Our study provides insights into mechanisms governing muscle repair promoted by exercise that will hopefully contribute towards better therapeutic interventions preventing sarcopenia.

## Ethics statement

The authors of this manuscript certify that they comply with the ethical guidelines for authorship and publishing in the *Journal of Cachexia, Sarcopenia and Muscle*.^68^

## Conflict of interest

None declared.

## Funding

This research was supported by the Fundação de Amparo à Pesquisa do Estado de São Paulo (FAPESP), Grant Number 2016/18633‐8, Conselho Nacional de Pesquisa e Desenvolvimento (CNPq) Grant Number 440436/2014, Coordenação de Aperfeiçoamento de Pessoal de Nível Superior (CAPES) Finance Code 001, and Centro de Pesquisa, Inovação e Difusão de Processos Redox em Biomedicina—CEPID Redoxoma, Grant 2013/07937‐8.

## Author contributions

Phablo Abreu and Alicia J. Kowaltowski contributed in the conceptualization of the study; Phablo Abreu and Alicia J. Kowaltowski in the data curation; Phablo Abreu in the formal analysis; Phablo Abreu and Alicia J. Kowaltowski in the funding acquisition; Phablo Abreu and Alicia J. Kowaltowski in the methodology; Phablo Abreu and Alicia J. Kowaltowski in the project administration; Alicia J. Kowaltowski in the supervision; Phablo Abreu and Alicia J. Kowaltowski in the writing of the original draft; and Phablo Abreu and Alicia J. Kowaltowski in writing of the review and editing.

## Supporting information


**Table S1.** Genes and primers

## References

[1] Yin H , Price F , Rudnicki MA . Satellite cells and the muscle stem cell niche. Physiol Rev 2013;93:23–67. 23303905 10.1152/physrev.00043.2011 PMC4073943

[2] von Maltzahn J , Jones AE , Parks RJ , Rudnicki MA . Pax7 is critical for the normal function of satellite cells in adult skeletal muscle. Proc Natl Acad Sci U S A 2013;110:16474–16479. 24065826 10.1073/pnas.1307680110 PMC3799311

[3] Bazgir B , Fathi R , Rezazadeh Valojerdi M , Mozdziak P , Asgari A . Satellite cells contribution to exercise mediated muscle hypertrophy and repair. Cell J 2017;18:473–484. 28042532 10.22074/cellj.2016.4714 PMC5086326

[4] Shea KL , Xiang W , LaPorta VS , Licht JD , Keller C , Basson MA , et al. Sprouty1 regulates reversible quiescence of a self‐renewing adult muscle stem cell pool during regeneration. Cell Stem Cell 2010;6:117–129. 20144785 10.1016/j.stem.2009.12.015 PMC2846417

[5] Chakkalakal JV , Jones KM , Basson MA , Brack AS . The aged niche disrupts muscle stem cell quiescence. Nature 2012;490:355–360. 23023126 10.1038/nature11438 PMC3605795

[6] Bigot A , Duddy WJ , Ouandaogo ZG , Negroni E , Mariot V , Ghimbovschi S , et al. Age‐associated methylation suppresses SPRY1, leading to a failure of re‐quiescence and loss of the reserve stem cell pool in elderly muscle. Cell Rep 2015;13:1172–1182. 26526994 10.1016/j.celrep.2015.09.067

[7] Christopher RR , Bjornson TH , Cheung LL , Tripathi PV , Steeper KM , Rando TA . Notch signaling is necessary to maintain quiescence in adult muscle stem cells. Stem Cells 2012;30:232–242. 22045613 10.1002/stem.773 PMC3384696

[8] Wen Y , Bi P , Liu W , Asakura A , Keller C , Kuang S . Constitutive Notch activation upregulates Pax7 and promotes the self‐renewal of skeletal muscle satellite cells. Mol Cell Biol 2012;32:2300–2311. 22493066 10.1128/MCB.06753-11 PMC3372272

[9] Gopinath SD , Webb AE , Brunet A , Rando TA . FOXO3 promotes quiescence in adult muscle stem cells during the process of self‐renewal. Stem Cell Reports 2014;2:414–426. 24749067 10.1016/j.stemcr.2014.02.002 PMC3986584

[10] Liu L , Charville GW , Cheung TH , Yoo B , Santos PJ , Schroeder M , et al. Impaired Notch signaling leads to a decrease in p53 activity and mitotic catastrophe in aged muscle stem cells. Cell Stem Cell 2018;23:544–556. 30244867 10.1016/j.stem.2018.08.019 PMC6173623

[11] Fujimaki S , Seko D , Kitajima Y , Yoshioka K , Tsuchiya Y , Masuda S , et al. Notch1 and Notch2 coordinately regulate stem cell function in the quiescent and activated states of muscle satellite cells. Stem Cells 2018;36:278–285. 29139178 10.1002/stem.2743

[12] Darr KC , Schultz E . Exercise‐induced satellite cell activation in growing and mature skeletal muscle. J Appl Physiol 1987;63:1816–1821. 3693217 10.1152/jappl.1987.63.5.1816

[13] Mangan G , Bombardier E , Mitchell AS , Quadrilatero J , Tiidus PM . Oestrogen‐dependent satellite cell activation and proliferation following a running exercise occurs via the PI3K signalling pathway and not IGF‐1. Acta Physiol (Oxf) 2014;212:75–85. 24862866 10.1111/apha.12317

[14] Abreu P , Mendes SV , Ceccatto VM , Hirabara SM . Satellite cell activation induced by aerobic muscle adaptation in response to endurance exercise in humans and rodents. Life Sci 2017;170:33–40. 27888112 10.1016/j.lfs.2016.11.016

[15] Jones AM , Carter H . The effect of endurance training on parameters of aerobic fitness. Sports Med 2000;29:373–386. 10870864 10.2165/00007256-200029060-00001

[16] Abreu P . Bioenergetics mechanisms regulating muscle stem cell self‐renewal commitment and function. Biomed Pharmacother 2018;103:463–472. 29674282 10.1016/j.biopha.2018.04.036

[17] Kurosaka M , Naito H , Ogura Y , Kojima A , Goto K , Katamoto S . Effects of voluntary wheel running on satellite cells in the rat plantaris muscle. J Sports Sci Med 2009;8:51–57. 24150556 PMC3737783

[18] Kurosaka M , Naito H , Ogura Y , Machida S , Katamoto S . Satellite cell pool enhancement in rat plantaris muscle by endurance training depends on intensity rather than duration. Acta Physiol 2012;205:159–166. 10.1111/j.1748-1716.2011.02381.x 22040028

[19] Shefer G , Rauner G , Yablonka‐Reuveni Z , Benayahu D . Reduced satellite cell numbers and myogenic capacity in aging can be alleviated by endurance exercise. PLoS One 2010;5:e13307. 20967266 10.1371/journal.pone.0013307 PMC2953499

[20] Shefer G , Rauner G , Stuelsatz P , Benayahu D , Yablonka‐Reuveni Z . Moderate‐intensity treadmill running promotes expansion of the satellite cell pool in young and old mice. FEBS J 2013;280:4063–4073. 23464362 10.1111/febs.12228 PMC3711960

[21] Brett JO , Arjona M , Ikeda M , Quarta M , Morrée A , Egner IM , et al. Exercise rejuvenates quiescent skeletal muscle stem cells in old mice through restoration of Cyclin D1. Nature Metab 2020;56:11–27. 10.1038/s42255-020-0190-0 PMC7323974 32601609

[22] Joanisse S , Nederveen JP , Baker JM , Snijders T , Iacono C , Parise G . Exercise conditioning in old mice improves skeletal muscle regeneration. FASEB J 2016;30:3256–3268. 27306336 10.1096/fj.201600143RR

[23] Baar K , Wende AR , Jones TE , Marison M , Nolte LA , Chen M , et al. Adaptations of skeletal muscle to exercise: rapid increase in the transcriptional coactivator PGC‐1. FASEB J 2002;16:1879–1886. 12468452 10.1096/fj.02-0367com

[24] Balan E , Schwalm C , Naslain D , Nielens H , Francaux M , Deldicque L . Regular endurance exercise promotes fission, mitophagy, and oxidative phosphorylation in human skeletal muscle independently of age. Front Physiol 2019;10:1088. 31507451 10.3389/fphys.2019.01088 PMC6713923

[25] Tang AH , Rando TA . Induction of autophagy supports the bioenergetic demands of quiescent muscle stem cell activation. EMBO J 2014;33:2782–2797. 25316028 10.15252/embj.201488278 PMC4282556

[26] Rooyackers OE , Adey DB , Ades PA , Nair KS . Effect of age on in vivo rates of mitochondrial protein synthesis in human skeletal muscle. Proc Natl Acad Sci U S A 1996;93:15364–15369. 8986817 10.1073/pnas.93.26.15364 PMC26410

[27] Conley KE , Jubrias SA , Esselman PC . Oxidative capacity and ageing in human muscle. J Physiol 2000;526:203–210. 10878112 10.1111/j.1469-7793.2000.t01-1-00203.x PMC2269983

[28] Chabi B , Ljubicic V , Menzies KJ , Huang JH , Saleem A , Hood DA . Mitochondrial function and apoptotic susceptibility in aging skeletal muscle. Aging Cell 2008;7:2–12. 18028258 10.1111/j.1474-9726.2007.00347.x

[29] Larsen S , Hey‐Mogensen M , Rabøl R , Stride N , Helge JW , Dela F . The influence of age and aerobic fitness: effects on mitochondrial respiration in skeletal muscle. Acta Physiol (Oxf) 2012;205:423–432. 22212519 10.1111/j.1748-1716.2012.02408.x

[30] Khacho M , Slack RS . Mitochondrial activity in the regulation of stem cell self‐renewal and differentiation. Curr Opin Cell Biol 2017;49:1–8. 29175320 10.1016/j.ceb.2017.11.003

[31] Forni MF , Peloggia J , Trudeau K , Shirihai O , Kowaltowski AJ . Murine mesenchymal stem cell commitment to differentiation is regulated by mitochondrial dynamics. Stem Cells 2016;34:743–755. 26638184 10.1002/stem.2248 PMC4803524

[32] Ferreira JC , Rolim NP , Bartholomeu JB , Gobatto CA , Kokubun E , Brum PC . Maximal lactate steady state in running mice: effect of exercise training. Clin Exp Pharmacol Physiol 2007;34:760–765. 17600553 10.1111/j.1440-1681.2007.04635.x

[33] Abreu P , Monteiro ICCR , Lima TA , dos Santos ACC , Ceccatto VM . Prescription of aerobic exercise training based on the incremental load test: a model of anaerobic threshold for rats. J Exerc Physiol 2012;15:47–52.

[34] Abreu P , Vitzel KF , Monteiro IC , Lima TI , Queiroz AN , Leal‐Cardoso JH , et al. Effects of endurance training on reduction of plasma glucose during high intensity constant and incremental speed tests in Wistar rats. Braz J Med Biol Res 2016;49:e5226. 27783805 10.1590/1414-431X20165226 PMC5089229

[35] Campos JC , Queliconi BB , Bozi LHM , Bechara LRG , Dourado PMM , Andres AM , et al. Exercise reestablishes autophagic flux and mitochondrial quality control in heart failure. Autophagy 2017;13:1304–1317. 28598232 10.1080/15548627.2017.1325062 PMC5584854

[36] Medeiros A , Oliveira EM , Gianolla R , Casarini DE , Negrao CE , Brum PC . Swimming training increases cardiac vagal activity and induces cardiac hypertrophy in rats. Braz J Med Biol Res 2004;37:1909–1917. 15558199 10.1590/s0100-879x2004001200018

[37] Junqueira LC , Bignolas G , Brentani RR . Picrosirius staining plus polarization microscopy, a specific method for collagen detection in tissue sections. Histochem J 1979;11:447–455. 91593 10.1007/BF01002772

[38] Conboy IM , Conboy MJ , Smythe GM , Rando TA . Notch‐mediated restoration of regenerative potential to aged muscle. Science 2003;302:1575–1577. 14645852 10.1126/science.1087573

[39] Sherwood RI , Christensen JL , Conboy IM , Conboy MJ , Rando TA , Weissman IL , et al. Isolation of adult mouse myogenic progenitors: functional heterogeneity of cells within and engrafting skeletal muscle. Cell 2004;119:543–554. 15537543 10.1016/j.cell.2004.10.021

[40] Cerletti M , Jurga S , Witczak CA , Hirshman MF , Shadrach JL , Goodyear LJ , et al. Highly efficient, functional engraftment of skeletal muscle stem cells in dystrophic muscles. Cell 2008;134:37–47. 18614009 10.1016/j.cell.2008.05.049 PMC3665268

[41] Gharaibeh B , Lu A , Tebbets J , Zheng B , Feduska J , Crisan M , et al. Isolation of a slowly adhering cell fraction containing stem cells from murine skeletal muscle by the preplate technique. Nat Protoc 2008;3:1501–1509. 18772878 10.1038/nprot.2008.142

[42] Cerqueira FM , Chausse B , Baranovski BM , Liesa M , Lewis EC , Shirihai OS , et al. Diluted serum from calorie‐restricted animals promotes mitochondrial β‐cell adaptations and protect against glucolipotoxicity. FEBS J 2016;283:822–833. 26732506 10.1111/febs.13632

[43] Hellemans J , Mortier G , De Paepe A , Speleman F , Vandesompele J . qBase relative quantification framework and software for management and automated analysis of real‐time quantitative PCR data. Genome Biol 2007;8:R19. 17291332 10.1186/gb-2007-8-2-r19 PMC1852402

[44] Speakman JR . Measuring energy metabolism in the mouse—theoretical, practical, and analytical considerations. Front Physiol 2013;4:34. 23504620 10.3389/fphys.2013.00034 PMC3596737

[45] Fan W , Waizenegger W , Lin CS , Sorrentino V , He MX , Wall CE , et al. PPARδ promotes running endurance by preserving glucose. Cell Metab 2017;25:1186–1193. 28467934 10.1016/j.cmet.2017.04.006 PMC5492977

[46] Cisterna B , Giagnacovo M , Costanzo M , Fattoretti P , Zancanaro C , Pellicciari C , et al. Adapted physical exercise enhances activation and differentiation potential of satellite cells in the skeletal muscle of old mice. J Anat 2016;228:771–783. 26739770 10.1111/joa.12429 PMC4831340

[47] Sacco A , Doyonnas R , Kraft P , Vitorovic S , Blau HM . Self‐renewal and expansion of single transplanted muscle stem cells. Nature 2008;456:502–506. 18806774 10.1038/nature07384 PMC2919355

[48] Ryall JG , Dell'Orso S , Derfoul A , Juan A , Zare H , Feng X , et al. The NAD^+^‐dependent SIRT1 deacetylase translates a metabolic switch into regulatory epigenetics in skeletal muscle stem cells. Cell Stem Cell 2015;16:171–183. 25600643 10.1016/j.stem.2014.12.004 PMC4320668

[49] Myers MJ , Shepherd DL , Durr AJ , Stanton DS , Mohamed JS , Hollander JM , et al. The role of SIRT1 in skeletal muscle function and repair of older mice. J Cachexia Sarcopenia Muscle 2019;10:929–949. 31197980 10.1002/jcsm.12437 PMC6711423

[50] Yang X , Yang S , Wang C , Kuang S . The hypoxia‐inducible factors HIF1α and HIF2α are dispensable for embryonic muscle development but essential for postnatal muscle regeneration. J Biol Chem 2017;292:5981–5991. 28232488 10.1074/jbc.M116.756312 PMC5392588

[51] Cerletti M , Jang YC , Finley LW , Haigis MC , Wagers AJ . Short‐term calorie restriction enhances skeletal muscle stem cell function. Cell Stem Cell 2012;10:515–519. 22560075 10.1016/j.stem.2012.04.002 PMC3561899

[52] Rodgers JT , King KY , Brett JO , Cromie MJ , Charville GW , Maguire KK , et al. mTORC1 controls the adaptive transition of quiescent stem cells from G_0_ to G_Alert_ . Nature 2014;510:393–396. 24870234 10.1038/nature13255 PMC4065227

[53] Zhang H , Ryu D , Wu Y , Gariani K , Wang X , Luan P , et al. NAD^+^ repletion improves mitochondrial and stem cell function and enhances life span in mice. Science 2016;352:1436–1443. 27127236 10.1126/science.aaf2693

[54] Coller HA . The paradox of metabolism in quiescent stem cells. FEBS Lett 2019;593:2817–2839. 31531979 10.1002/1873-3468.13608 PMC7034665

[55] Wilkinson SB , Phillips SM , Atherton PJ , Patel R , Yarasheski KE , Tarnopolsky MA , et al. Differential effects of resistance and endurance exercise in the fed state on signalling molecule phosphorylation and protein synthesis in human muscle. J Physiol 2008;586:3701–3717. 18556367 10.1113/jphysiol.2008.153916 PMC2538832

[56] Hawley JA , Hargreaves M , Joyner MJ , Zierath JR . Integrative biology of exercise. Cell 2014;159:738–749. 25417152 10.1016/j.cell.2014.10.029

[57] Lanza IR , Short DK , Short KR , Raghavakaimal S , Basu R , Joyner MJ , et al. Endurance exercise as a countermeasure for aging. Diabetes 2008;57:2933–2942. 18716044 10.2337/db08-0349 PMC2570389

[58] Molé PA , Oscai LB , Holloszy JO . Adaptation of muscle to exercise. Increase in levels of palmityl CoA synthetase, carnitine palmityltransferase, and palmityl CoA dehydrogenase, and in the capacity to oxidize fatty acids. J Clin Invest 1971;50:2323–2330. 5096516 10.1172/JCI106730 PMC292174

[59] Holloszy JO , Booth FW . Biochemical adaptations to endurance exercise in muscle. Annu Rev Physiol 1976;38:273–291. 130825 10.1146/annurev.ph.38.030176.001421

[60] Overmyer KA , Evans CR , Qi NR , Minogue CE , Carson JJ , Chermside‐Scabbo CJ , et al. Maximal oxidative capacity during exercise is associated with skeletal muscle fuel selection and dynamic changes in mitochondrial protein acetylation. Cell Metab 2015;21:468–478. 25738461 10.1016/j.cmet.2015.02.007 PMC4350023

[61] Paulsen G , Egner I , Raastad T , Reinholt F , Owe S , Lauritzen F , et al. Inflammatory markers CD11b, CD16, CD66b, CD68, myeloperoxidase and neutrophil elastase in eccentric exercised human skeletal muscles. Histochem Cell Biol 2013;139:691–715. 23224298 10.1007/s00418-012-1061-x

[62] Siles L , Ninfali C , Cortés M , Darling DS , Postigo A . ZEB1 protects skeletal muscle from damage and is required for its regeneration. Nat Commun 2019;10:1364. 30910999 10.1038/s41467-019-08983-8 PMC6434033

[63] Feige P , Brun CE , Ritso M , Rudnicki MA . Orienting muscle stem cells for regeneration in homeostasis, aging, and disease. Cell Stem Cell 2018;23:653–664. 30388423 10.1016/j.stem.2018.10.006 PMC6262894

[64] Forcina L , Miano C , Pelosi L , Musarò A . An overview about the biology of skeletal muscle satellite cells. Curr Genomics 2019;20:24–37. 31015789 10.2174/1389202920666190116094736 PMC6446479

[65] Varum S , Rodrigues AS , Moura MB , Momcilovic O , Easley CA 4th , Ramalho‐Santos J , et al. Energy metabolism in human pluripotent stem cells and their differentiated counterparts. PLoS One. 2011;6:e20914. 21698063 10.1371/journal.pone.0020914 PMC3117868

[66] Xu X , Duan S , Yi F , Ocampo A , Liu GH , Izpisua Belmonte JC . Mitochondrial regulation in pluripotent stem cells. Cell Metab 2013;18:325–332. 23850316 10.1016/j.cmet.2013.06.005

[67] de Carvalho AETS , Bassaneze V , Forni MF , Keusseyan AA , Kowaltowski AJ , Krieger JE . Early postnatal cardiomyocyte proliferation requires high oxidative energy metabolism. Sci Rep 2017;7:15434. 29133820 10.1038/s41598-017-15656-3 PMC5684334

[68] von Haehling S , Morley JE , Coats AJS , Anker SD . Ethical guidelines for publishing in the Journal of Cachexia, Sarcopenia and Muscle: update 2019. J Cachexia Sarcopenia Muscle 2019;10:1143–1145. 31661195 10.1002/jcsm.12501 PMC6818444

