# Supplementary material for: Satellite cell self‐renewal in endurance exercise is mediated by inhibition of mitochondrial oxygen consumption
Source: J Cachexia Sarcopenia Muscle. 2020 Aug 3;11(6):1661–76. doi: 10.1002/jcsm.12601 (PMC7749620; doi:10.1002/jcsm.12601)
Supplement: Supplementary file 1 — Table S1. Genes and primers [file JCSM-11-1661-s001.docx]

Supplementary Table 1: Genes and primers

| **Gene** | **Primer Bank ID** | **Forward Primer** | **Reverse Primer** |
| --- | --- | --- | --- |
| ND1 (mtDNA) | NC_012920.1 | ACTACGCAAAGGCCCCAACG | GAGCTAAGGTCGGGGCGGTG |
| HPRT | NC_000023.11 | TGACATGTGCCGCCTGCGAG | GTGGTCGCTTTCCGTGCCGA |
| CXCR4 | 116268122c1 | GACTGGCATAGTCGGCAATG | AGAAGGGGAGTGTGATGACAAA |
| MYOD1 | 6996932a1 | CCACTCCGGGACATAGACTTG | AAAAGCGCAGGTCTGGTGAG |
| NOTCH1 | 13177625a1 | GATGGCCTCAATGGGTACAAG | TCGTTGTTGTTGATGTCACAGT |
| SPRY1 | 24657551a1 | ATGGATTCCCCAAGTCAGCAT | CCTGTCATAGTCTAACCTCTGCC |
| SIRT1 | 9790229a1 | GCTGACGACTTCGACGACG | TCGGTCAACAGGAGGTTGTCT |
| NAMPT | 10946948a1 | GCAGAAGCCGAGTTCAACATC | TTTTCACGGCATTCAAAGTAGGA |
| OPA1 | 19526960a1 | TGGAAAATGGTTCGAGAGTCAG | CATTCCGTCTCTAGGTTAAAGCG |
| MFN1 | 28436944a1 | CCTACTGCTCCTTCTAACCCA | AGGGACGCCAATCCTGTGA |
| MFN2 | 120407047c1 | AGAACTGGACCCGGTTACCA | CACTTCGCTGATACCCCTGA |
| DRP1 | 22779912a1 | CAGGAATTGTTACGGTTCCCTA | CCTGAATTAACTTGTCCCGTGA |
| FIS1 | 13384998a1 | TGTCCAAGAGCACGCAATTTG | CCTCGCACATACTTTAGAGCCTT |
| HEY1 | 6754188a1 | GCGCGGACGAGAATGGAAA | TCAGGTGATCCACAGTCATCTG |
| HES1 | 6680205a1 | CCAGCCAGTGTCAACACGA | AATGCCGGGAGCTATCTTTCT |
| RBPJ | 13385336a1 | GGGAAGTCATTCGCCAATTTCA | CACGTTCATCTCCCATAAGCAAT |
| FOXO3A | 9789951a1 | CTGGGGGAACCTGTCCTATG | TCATTCTGAACGCGCATGAAG |
| HIF1 | 28892777a1 | GTCCCAGCTACGAAGTTACAGC | CAGTGCAGGATACACAAGGTTT |
| TFAM | 1575501a1 | ATTCCGAAGTGTTTTTCCAGCA | TCTGAAAGTTTTGCATCTGGGT |
| PGC1 | 29244579a1 | ATGAAGAGTATCCGGGAGACC | TGGGCTCATAGAGTACACTGTAG |
| Myogenin | 13654247a1 | GAGACATCCCCCTATTTCTACCA | GCTCAGTCCGCTCATAGCC |
